# SVIP regulates Z variant alpha-1 antitrypsin retro-translocation by inhibiting ubiquitin ligase gp78

**DOI:** 10.1371/journal.pone.0172983

**Published:** 2017-03-16

**Authors:** Nazli Khodayari, Rejean liqun Wang, George Marek, Karina Krotova, Mariana Kirst, Chen Liu, Farshid Rouhani, Mark Brantly

**Affiliations:** 1 Division of Pulmonary, Critical Care and Sleep Medicine, Department of Medicine, University of Florida, Gainesville, Florida, United States; 2 Department of Pathology and Laboratory Medicine, Rutgers University, New Brunswick, New Jersey, United States; University of British Columbia, CANADA

## Abstract

Alpha-1 antitrypsin deficiency (AATD) is an inherited disorder characterized by early-onset emphysema and liver disease. The most common disease-causing mutation is a single amino acid substitution (Glu/Lys) at amino acid 342 of the mature protein, resulting in disruption of the 290–342 salt bridge (an electrophoretic abnormality defining the mutation [Z allele, or ZAAT]), protein misfolding, polymerization, and accumulation in the endoplasmic reticulum of hepatocytes and monocytes. The Z allele causes a toxic gain of function, and the E3 ubiquitin ligase gp78 promotes degradation and increased solubility of endogenous ZAAT. We hypothesized that the accumulation of ZAAT is influenced by modulation of gp78 E3 ligase and SVIP (small VCP-interacting protein) interaction with p97/VCP in ZAAT-expressing hepatocytes. We showed that the SVIP inhibitory effect on ERAD due to overexpression causes the accumulation of ZAAT in a human Z hepatocyte–like cell line (AT01). Overexpression of gp78, as well as SVIP suppression, induces gp78-VCP/p97 interaction in AT01 cells. This interaction leads to retro-translocation of ZAAT and reduction of the SVIP inhibitory role in ERAD. In this context, overexpression of gp78 or SVIP suppression may eliminate the toxic gain of function associated with polymerization of ZAAT, thus providing a potential new therapeutic approach to the treatment of AATD.

## Introduction

Alpha-1 antitrypsin (AAT), is a 52-KD globular protein mostly produced in hepatocytes. AAT is the most abundant serum serine protease inhibitor exerting its neutrophil elastase-neutralizing action throughout the body and, in particular, in the lung [1, 2]. Serum AAT deficiency (AATD), is an autosomal recessive metabolic disorder, which there is a deficiency in the concentration of circulating AAT. AATD has been associated with hereditary early-onset emphysema [3]. Several AAT genetic variants have been associated with disease inheritance, the most common variant being a Glu to Lys mutation in position 342 (Glu342Lys, or ZAAT). ZAAT occurs in one in 2000 live births, and homozygous carriage is associated with serum protease inhibitor (PI) deficiency and early and severe lung disease [4]. In addition, AATD is the most common genetic cause of liver disease in children; exaggerated amounts of ZAAT polymers accumulate in the liver, causing liver inflammation and fibrosis and, eventually, cirrhosis [5–7]. The Glu342Lys variant, or ZAAT, is the result of the formation of a salt bridge between Glu342 and Lys290, leading to a reactive loop insertion from one molecule into the β-sheet of a second molecule and aberrant folding followed by polymer formation [8–10]. As a result, ZAAT polymers accumulate in the endoplasmic reticulum (ER) of hepatocytes, resulting in low plasma concentrations of functional AAT, leading to emphysema and liver damage [11].

The ability of a cell to maintain quality control of misfolded proteins is critical for cellular vitality [12]. The accumulation of misfolded proteins is often toxic to the cells and directly related to cellular injury, which has been seen in such diseases as AATD [13]. Although ER stress and ER-associated degradation (ERAD) mechanisms are believed to be important in the processing of ZAAT and development of liver disease, the complete mechanisms underlying ZAAT polymerization and degradation have not been fully elucidated [11]. The ER of hepatocytes is equipped with a quality control system, which includes the molecular chaperones and folding sensors that detect correctly folded proteins and export them from the ER to their final destination or retain and refold misfolded proteins [14]. When ER quality control system fails to refold folding intermediates and misfolded proteins, cells activate ERAD. ERAD is a secondary defensive mechanism [15, 16] maintaining homeostasis in the Golgi secretory pathway [17] by retro-transporting misfolded proteins from the ER into the cytoplasm, where they are ubiquitinated for proteasomal degradation [18, 19]. ERAD requires coordinated retro-translocation (extraction) through pore proteins within the ER membrane, ubiquitination, and degradation by proteasomes. ERAD E3 ligase gp78 (also known as tumor autocrine motility factor, or AMFR) is one of the core components of protein degradation in ERAD [20]. gp78 is largely localized to the ER membrane and has the capacity to target well-characterized ERAD substrates, including ZAAT [21]. The knockdown of gp78 by siRNA abolishes ERAD in several mammalian ERAD substrates, including ZAAT, suggesting gp78-mediated ubiquitination is an early event in the process of retro-translocation [22].

p97/VCP, a member of the AAA (ATPase associated with various cellular activities) ATPase family, participates in protein degradation through interaction with a large number of partners and protein cofactors, such as gp78. The interaction between p97/VCP and gp78 enhances the binding of p97/VCP to polyubiquitinated proteins, such as ZAAT [23, 24]. Recent studies specify a role for p97/VCP in extracting polypeptides from the ER membrane [25, 26]. p97/VCP interacts with gp78 E3 ligase through its VCP-interacting motif (VIM) [27, 28].

The highly conserved VIM is important for interaction with p97/VCP. gp78 has a VIM, which allows the two partners to complete the cycle of retro-translocation and ubiquitination. In 2002, Nagahama et al. identified a small p97/VCP-interacting protein (SVIP), which contains the same VIM domain. SVIP has 76 amino acids with two putative coiled-coil regions [29]. SVIP shares the VIM motif with gp78 and may compete with the E3 ligase binding to p97/VCP to regulate VCP function [30, 31]. The negative regulatory role of SVIP in ERAD has been shown by formation of vacuoles, which may be caused by accumulation of misfolded proteins, when SVIP is overexpressed [32]. The ERAD inhibiting role of SVIP is reduced through SVIP protein downregulation during ER stress. However, prolonged ER stress results in the accumulation of misfolded protein in the ER and significantly upregulates SVIP [33].

This study has been designed to investigate whether SVIP has a regulatory role in gp78-mediated ERAD in hepatocytes with ZAAT aggregation. The data indicate that SVIP regulates the formation of a gp78-p97/VCP complex, thereby changing ZAAT ERAD efficacy. Furthermore, SVIP is upregulated in the presence of ZAAT and inhibits ERAD through gp78-p97/VCP interaction, leading to increased ZAAT accumulation and AATD-associated liver disease.

## Material and methods

### Reagents and antibodies

Primers, Dynabeads protein A, Superscript VILO cDNA synthesis kit, VCP siRNA, and SVIP siRNA were purchased from Life Technologies (Carlsbad, CA), and X-tremeGENE siRNA and, HP DNA transfection reagent were purchased from Roche Applied Science (Indianapolis, IN). The rabbit polyclonal antibodies against AMFR and ubiquitin and anti-rabbit IgG (conformation specific) were purchased from Cell Signaling (Danvers, MA). The rabbit polyclonal antibody against N-terminus AMFR was purchased from LifeSpan BioScience (Seattle, WA). Polyclonal anti-rabbit antibody against SVIP and mouse monoclonal antibody against actin were purchased from Sigma-Aldrich (St. Louis, MO) Polyclonal anti-rabbit antibodies against p97/VCP and IP lysis buffer were purchased from Thermo Fisher scientific (Waltham, MA). Polyclonal anti-rabbit antibody against AAT was purchased from Dako (Carpentaria, CA).

### Human tissue and cell line establishment

Anonymized human tissue for this study was obtained through Alpha-1 Foundation DNA and Tissue Bank, University of Florida IRB number 201500842, ClinicalTrials.gov identifier: NCT00884455. Samples where anonymized by Bank personal and then disturbed to the researchers. The reported study was specifically approved by University of Florida IRB#201700065 and entitled Molecular Mechanisms of Alpha-1-Antitrypsin Accumulation in the Liver. Written consent was obtained from all study subjects enrolled the Alpha-1 Foundation DNA and Tissue Bank. The Bank and UF IRB assure that all samples were obtained according to the principles expressed in the Declaration of Helsinki. Three cell lines were initially included in the present study. Our group developed two of the cell lines: AT01 and Hu339. The AT01 (PiZZ) and Hu339 (PiMM) cell lines was derived from human liver tissue. The cells were immortalized with infection of adenovirus-expressing human telomerase reverse transcriptase (hTERT) gene [34, 35]. However, these cells have a low level of AAT expression and accumulation that was supplemented in the present study through in vitro transfection. The plasmids used in the transient transfection harbor the MAAT (normal variant) or ZAAT gene tagged with red flourscent protein (RFP). The third cell line used in this study is a well-characterized, hepatocyte-derived cellular carcinoma cell line, Huh 7.5 cells (generous gift of Dr. Chen Liu’s lab).

Hepatocyte-like MM cell line (Hu339) and hepatocyte-like ZZ cell line (AT01) were cultured in DMEM/F12 with 10% bovine serum and 5% penicillin/streptomycin and were checked for liver cell gene markers, such as alpha fetal protein (AFP), albumin, and AAT. Cells were transiently transfected with the indicated plasmids by the X-TremeGENE transfection reagent, according to the manufacturer’s instructions. Stably-transfected Huh 7.5 cells were generated by selecting for successful integration of the RFP-ZAAT gene using the antibiotic Geneticin. Selection occurs in a 10-cm tissue culture plate, where it takes approximately 2–3 weeks for foci of Geneticin-resistant colonies to appear, after which the resistant foci are clonally expanded over a period of approximately 2–3 weeks.

### Ethics statement

Anonymized human tissue for this study was obtained through Alpha-1 Foundation DNA and Tissue Bank, University of Florida IRB number 201500842, ClinicalTrials.gov identifier: NCT00884455. Samples where anonymized by Bank personal and then disturbed to the researchers. The reported study was specifically approved by University of Florida IRB#201700065 and entitled Molecular Mechanisms of Alpha-1-Antitrypsin Accumulation in the Liver. Written consent was obtained from all study subjects enrolled the Alpha-1 Foundation DNA and Tissue Bank. The Bank and UF IRB assure that all samples were obtained according to the principles expressed in the Declaration of Helsinki.

### Creation of CRISPR knockout for AAT gene

CRISPR (clustered regulatory interspaced short palindromic repeats) knockouts were created as described previously [36]. Briefly, Huh 7.5 cells were transiently transfected with 2 μg per six-well of pSpCas9(BB)-2A-GFP encoding Cas9 and small guide RNA against AAT, using the X-tremeGENE HP DNA transfection reagent. After 24 hours, transfected cells were diluted into 10-mm dishes and grown for another week and then single-cell cloned. AAT knockout clones were identified using ELISA and Western blotting using polyclonal anti-rabbit antibody against AAT.

### Liver tissue samples used for immunoblotting

A total of six explanted liver tissue samples (three normal controls [PiMM] and three alpha-1 antitrypsin deficient individuals [PiZZ]) were used for Western blotting (see IRB protocols listed in Human Tissue and Cell Lines section). Pi stands for protease inhibitor. PiMM is the term for being homozygous for the normal AAT variant, and PiZZ stands for being homozygous for the ZAAT variant. Each sample was lysed using RIPA buffer containing 1:1000 phosphatase inhibitor cocktail and PI EDTA-free from Calbiochem (San Diego, CA). The concentration of protein lysates was determined by the Thermo Fisher scientific (Waltham, MA) protein assay kit, according to the manufacturer's instructions, using bovine albumin as a protein standard.

### cDNAs and constructs

The full-length cDNA of gp78 fragment was cut from pCMV6-Entry-AMFR plasmid (OriGene, Rockville, MD) by KpnI and NotI sites, which was sub-cloned into the same sites of pTR2-CB-MAAT vector (a kind gift from Dr. Shihong Song’s Lab at the University of Florida) to swap the MAAT gene. The plasmid was digested with Hind III and NotI and cloned into the same sites in pCR3.1 plasmid (Invitrogen, Carlsbad, CA) to form pCR3.1-gp78 plasmid. pCR3.1-T plasmid (Invitrogen) was self-ligated to form pCR3.1-empty plasmid. The gp 78 Δ135 amino acid fragment was obtained from PCR with primer pair: 5´AAGCTTATGCCGGTGCTCTTCCTCGA3´ and 5´ACGCGTCTAATCTGACCGCTGTGTAGGAA3´ to introduce stop codon TAG before C terminal 135 amino acid. The fragment was cloned into pCR3.1-GP78-3 Flag plasmid to swap GP78 fragment from HindIII and MluI sites to make pCR3.1-gp 78 Δ135. pCR3.1-EGFP-gp 78 was a gnerous gift from Dr. Allan M Weissman from the NIH (Bethesda, MD).

pCMV6-Entry-SVIP plasmid was purchased from OriGene Technologies, HA-Ubiquitin plasmid and pSpCas9(BB)-2A-GFP encoding Cas9 and sgRNA plasmid were purchased from AddGene (Cambridge, MA), and pCDNA3.1-SVIP mutant with five amino acid mutations (R22E, L25Q, A26V, R31D, R32E) and Myc tag and Flag tag on 3´ end was synthesized and cloned into pCDNA3.1 vector by Life Technologies. The ZAAT with mRFP cloned into BamH1 site was sub-cloned into Hind III and Not I sites of PCR3.1 plasmid (Invitrogen) to make the Z-RFP plasmid. The DNA sequence was confirmed by Sanger sequencing.

### Quantitative real-time PCR

The total RNA was extracted from liver tissue samples and selected cell lines, and the first complementary strand was generated. Expression level of SVIP and p97/VCP gene products were analyzed by quantitative real-time PCR using an Applied Biosystems 7500 fast Real-Time PCR system from Life Technologies and TaqMan Universal PCR Master Mix from Roche Applied Science. Pairs of genes were analyzed simultaneously, and h18S ribosomal RNA was used as an endogenous control. The specificity of the priming and amplification was verified with a melt curve for each amplicon. The quantitative real-time PCR was performed in duplicates, and results were averaged. Results are presented as the relative quantification (RQ) as determined by the 2^−ΔΔCt^ equation.

### Immunoblotting and immunoprecipitation

AT01 and Hu339 cells were seeded at 3 × 10^5^/well in 6-well plates 24 hours prior to the day of transfection with X-TremeGENE HP DNA transfection reagent (Roche, Basel, Switzerland) or electroporation (Lonza, Basel, Switzerland) (for plasmids) and X-TremeGENE siRNA transfection reagent (Roche) (for siRNAs). Transfected cells were collected 48 hours post transfection and lysed in RIPA buffer containing 50 mM Tris, 150 mM NaCl, 0.5% SDS, 0.5% sodium deoxycholate, and 1% NP-40. For immunoprecipitation (IP), lysates were incubated with 1 μg of antibody and 50 μL of Dynabeads protein A beads (Novex) for 2 hours at 4°C. Beads were washed three times in buffer containing 1× PBS and 0.1% Triton X-100 before processing for immunoblotting (IB). Total protein was resolved on Tris glycine sodium dodecyl sulfate-polyacrylamide gel electrophoresis (SDS-PAGE) gels (Bio-Rad, Hercules, CA). Proteins were transferred to nitrocellulose membranes. The blots were blocked and incubated with antibodies overnight at 4°C. The immunocomplex was washed three times and then incubated with either goat anti-mouse or goat anti-rabbit IgG IP-specific secondary antibodies (Abcam) at 1:1000 dilution for 1 hour at room temperature. Proteins were detected by SuperSignal West Dura Extended Duration Substrate Kit (Thermo Scientific).

### Analysis of ZAAT ubiquitination and degradation

AT01 cells were transfected with or without empty plasmid, WT gp78, gp78 Δ135, WT SVIP plasmid. After 48 hours, cells were incubated with 20 μM proteasome inhibitor MG132 for 6 hours and lysed in IP lysis buffer containing 10 mM iodoacetamide. Total ubiquitination was examined by Western blotting for polyubiquitin and actin as a loading control, and ZAAT ubiquitination was assessed by co-IP for ZAAT followed by SDS-PAGE and IB for ubiquitin.

### Transmission electron microscopy

PIZZ and PIMM cells were transfected with or without 22 μg WT SVIP plasmid or siSVIP and were preserved in 1% glutaraldehyde in Tyrode’s buffer, rinsed with Tyrode’s buffer, scraped from the culture dishes, and pelleted in centrifuge tubes. Pelleted cells were rinsed with 2-mercaptoethanol in 0.1M Na cacodylate buffer (2-ME buffer), post-fixed in 1% osmium tetroxide in 2-ME buffer, and washed in 2-ME buffer. The samples were dehydrated in a graded series of ethanols and propylene oxide and embedded in epoxy resin (Taab 812 Resin, Marivac Industries, Montreal, CA). Ultrathin (60–70 nm) sections were counterstained with uranyl acetate and lead citrate and observed using a Hitachi 7600 transmission electron microscope (Hitachi High-Technologies America, Schaumburg, IL) equipped with a Macrofire monochrome progressive scan CCD camera (Optronics, Goleta, CA) and AMTV image capture software (Advanced Microscopy Techniques, Danvers, MA).

### Metabolic labeling studies

Nearly confluent monolayers of AT01 cells were co-transfected with eukaryotic expression vectors containing Z-RFP and SVIP or empty plasmid in 100-mm diameter culture dishes. The cells were incubated for 30 minutes with [^35^S] methionine (200–500 μCi/ml of medium; PerkinElmers, Waltham, MA) [37] and then chased for 0–8 hours by incubation in 3 mL of RPMI with 10% fetal bovine serum containing a five-fold excess of unlabeled methionine. Cells were harvested in a total volume of 1 mL IP lysis buffer (Pierce, Waltham, MA), kept in dry ice, and centrifuged to precipitate cell debris. ZAAT-RFP was immunoprecipitated from cell lysate and medium using rabbit anti-human AAT antibody bound to protein A dynabeads. Imunocomplexes were washed, suspended in 20 μL of sample buffer, heated at 70°C for 10 min, and analyzed using SDS Tris-glysin 10% page (Bio-Rad). Radiolabeled AAT was detected by autoradiography.

### Immunostaining, immunofluorescence microscopy, and image analysis

AAT knock out cells were co-transfected with 1 μg Z-RFP and 2 μg empty vector, gp78-EGFP plasmid [21], SVIP, or siSVIP. The cells were grown on coverslips and were fixed with 4% paraformaldehyde. Cells were washed and mounted with slowfade diamond antifade mountant with DAPI (Life Technologies) on glass slides, and images were collected using a fluorescence microscope (Nikon, Inc., Melville, NY). Samples were scanned with a 0.1-μm step. Images were processed for brightness and contrast and filtered for noise with Volocity 6.3 software (Perkin Elmer, Waltham, MA) following good practices, as outlined by Rossner et al. [38].

### Statistical analysis

All results are expressed as mean ± S.E. Statistical analysis was performed using Prism 6 software program. Values of *p* < 0.05 was considered statistically significant.

## Results

### gp78 regulates degradation of ubiquitinated ZAAT and targets ZAAT for proteasomal degradation

**Fig 1:**
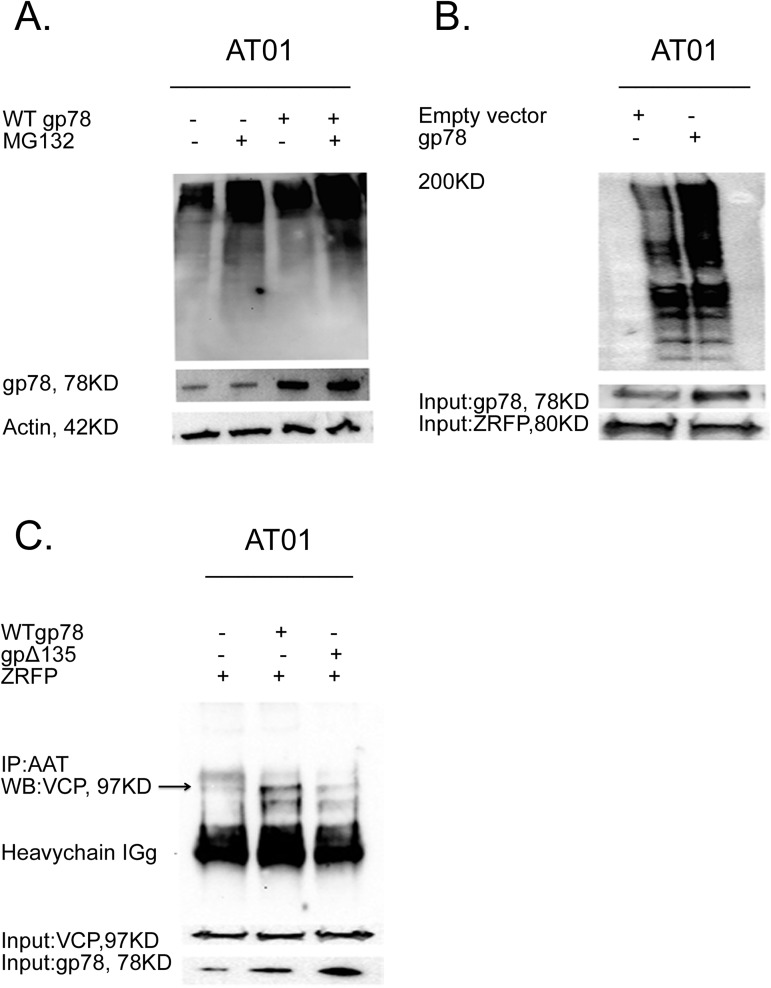
Gp78 targets ZAAT for proteasomal degradation. **(A) Changes in the accumulation** of polyubiquitinated proteins in presence of proteasome inhibitor MG132. (B) WT gp78 increases the level of ubiquitinated AAT. AT01 cells co-transfected with ZAAT and empty vector or WT gp78. At 24 hours post transfection, the cells were incubated with 20 nM MG132 for 6 hours and immunoprecipitated with AAT antibody, followed by WB with anti-ubiquitin rabbit polyclonal antibody. (C) WT gp78 enhances ZAAT-p97/VCP association through VIM domain. AAT was immunoprecipitated with AAT antibody from AT01 transfected with Z-RFP and with or without WT gp78 and gp78 Δ135. p97/VCP associated with AAT was determined by IB with anti-p97/VCP antibody.

gp78 enhances the interaction of misfolded proteins with p97/VCP and facilitates their degradation [27]. To assess the degradation facilitated by the overexpression of gp78, the level of total polyubiquitination was detected in AT01 cells overexpressing WT gp78 upon a 6-hour incubation with 20 nM proteasome inhibitor MG132 by Western blot using the ubiquitin antibody. MG132 caused a significant accumulation of total polyubiquitination in WT gp78-transfected cells as compared with the control AT01 cells. Actin was used as a loading control (Fig 1A).

To examine the AAT degradation facilitation by the overexpression of gp78, the level of ubiquitinated AAT was detected in AT01 cells co-transfected with 2 μg ZAAT plasmid and WT gp78. At 48 hours post transfection, cells were incubated with 20 μM proteasome inhibitor MG132 for 6 hours and subjected to co-IP for AAT, followed by WB using ubiquitin antibody. MG132 caused a significant accumulation of polyubiquitinated AAT in WT gp78-transfected cells as compared with control cells (Fig 1B). To investigate whether gp78 regulates the level of ZAAT and p97/VCP interaction, we analyzed the levels of ZAAT and p97/VCP binding in AT01 cells, which overexpressed WT gp78 or gp78Δ135. gp78Δ135 is a truncation of the C-terminal 135 amino acids of gp78, which leads to the loss of p97/VCP binding [27]. AT01 cells co-transfected with Z-RFP plasmid and plasmids encoding WT gp78 or gp78Δ135 were processed for IP for AAT, followed by WB with the p97/VCP antibody. Under these conditions, WT gp78 dramatically increased the level of interaction between AAT and p97/VCP (Fig 1C).

### Functional interactions between gp78 and p97/VCP in AATD

Proteins that fail to meet conformational standards, such as misfolded proteins, are selected and ultimately degraded by the ubiquitin-proteasome system of ERAD [39]. gp78 represents one of the core components for ubiquitination in a subset of misfolded substrates before their extraction from the ER membrane, including ZAAT [40]. Other components of ERAD, such as p97/VCP, are necessary for retro-translocation of these substrates. It has been recently reported that gp78 interacts with p97/VCP directly through the p97/VCP interacting motif (VIM) [30]. Previously, it had been reported that gp78 facilitates the solubility of exogenous ZAAT in HEK293 cells [23].

**Fig 2:**
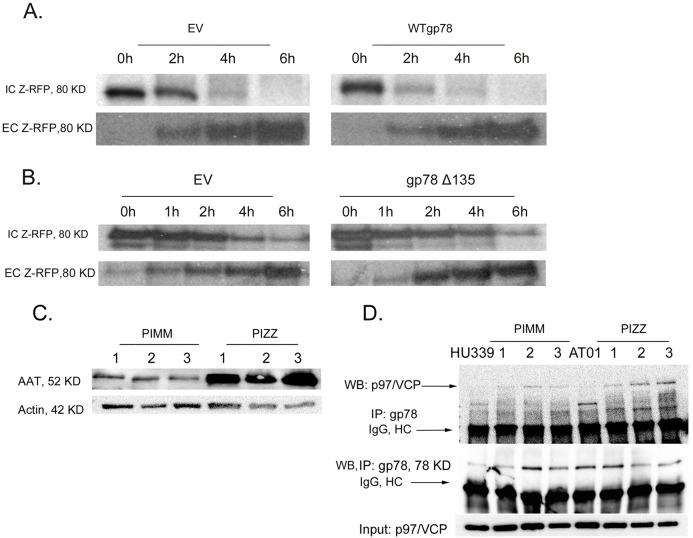
Functional interactions between gp78 and p97/VCP in AATD. (A) gp78 overexpression increased the level of ZAAT degradation in Huh 7.5 knockout cells stably expressing ZRFP. Cells were transfected with empty plasmid or WT gp78 as indicated. At 48 hours post transfection, cells were radiolabeled with 500 μCi ^35^S and chased for the indicated times. The levels of intracellular (IC) and extracellular (EC) AAT were determined by SDS-PAGE autoradiography. (B) gp78 enhanced ZAAT degradation through interaction with p97/VCP. Cells were transfected with empty plasmid or gp78Δ135 as indicated. Experiemtns were performed as in A. (C) Expression level of AAT in liver tissue samples. (D) ZAAT accumulation enhanced the gp78-VCP interaction. Co-IP was performed on Hu339 and AT01 cells and liver tissue samples of three healthy controls and three AATD individuals; 100 μg of the total lysate was processed for IP with anti-gp78, followed by WB with anti-gp78 and anti-p97/VCP to detect the level of gp78 and associated p97/VCP.

To assess whether gp78 had a role in the degradation of ZAAT, we transfected WT gp78 into Huh 7.5 knockout cells stably expressing RFP-ZAAT. At 48 hours post transfection, the cells were processed to determine the levels of ZAAT by pulse chase experiments. As compared with the control cells, gp78 overexpression significantly decreased the level of accumulated ZAAT and caused faster ZAAT clearance from the ER without significant changes in the level of extracellular ZAAT (Fig 2A). Then, we investigated whether p97/VCP is involved in gp78-mediated ERAD and ZAAT retro-translocation. To assess this question, empty plasmid or gp78Δ135 was transfected into the same sets of cells and ZAAT degradation was evaluated by pulse chase studies. Transfection with gp78Δ135 does not affect the level of intracellular and extracellular ZAAT as compared with transfection with empty plasmid (Fig 2B).

p97/VCP is a known chaperone that inhibits protein aggregation. To assess the association between gp78 and p97/VCP in control and deficient liver tissue samples, 10 μg of total lysate was subjected to IB to determine and confirm the high level of antitrypsin and ER stress in deficient samples (Fig 2C). AAT aggregated in three deficient liver samples, whereas this was not significant in normal controls. Then, we investigated whether the aggregation of ZAAT leads to enhanced gp78-p97/VCP interaction to gp78-mediated ERAD. Co-IP was performed for the total tissue lysates using gp78 antibody, followed by anti-gp78 as input and anti-p97/VCP antibody (Fig 2D). The results show enhanced gp78-p97/VCP interaction in three AAT-deficient liver tissue samples regardless of the basal gp78 expression level of the individuals (Fig 2D).

### SVIP is overexpressed in the cells under prolonged ER stress

**Fig 3:**
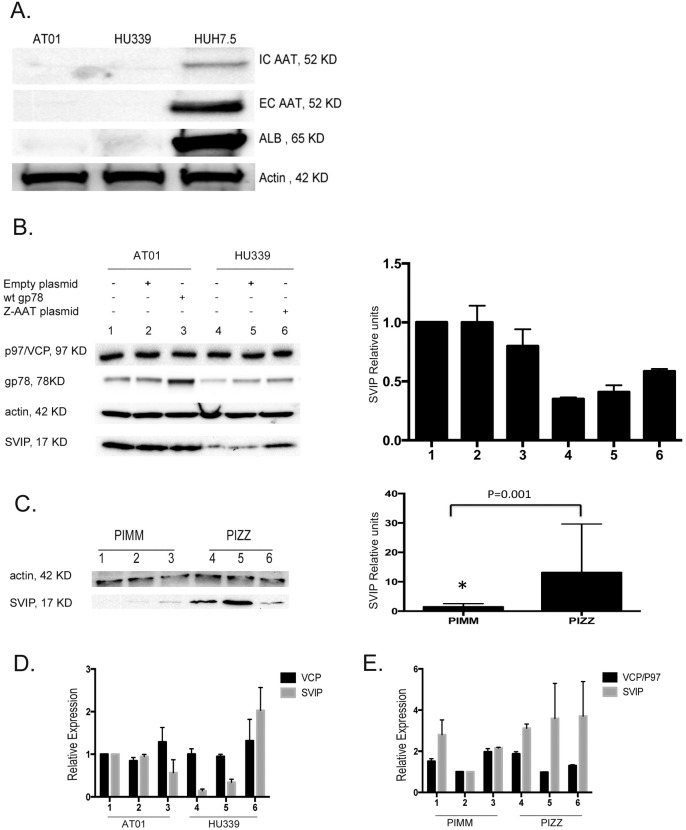
SVIP is overexpressed in cells under prolonged ER stress. (A) Western blot of intracellular and extracellular (EC) levels of AAT and albumin expression level in cell lines. AT01 and Hu339 cells were lysed in RIPA buffer, and protein concentration was measured using Pierce BCA protein assay kit; 10 μg total lysate was resolved on 10% TGX electrophoresis gel. The volume of the media to detect EC AAT was correlated to total protein in corresponding cell lysate. (B) Western blot of SVIP and VCP protein expression levels and relative densities in the AT01 cells under prolonged ER stress and in Hu339 cells transfected with ZAAT plasmid. Cells were transfected with WT gp78 or pTR2-CB-ZAAT, as indicated. At 48 hours post transfection, total lysates were subjected to Western blot analysis. β-Actin levels were measured to demonstrate equal sample loading. (C) SVIP protein expression level and relative units in deficient and normal liver tissue samples. (D) SVIP mRNA expression as analyzed by quantitative PCR in cell lines AT01 and Hu339, with same treatments as panel B, and (E) in the liver tissue samples, respectively.

AT01 (PiZZ genotype) and Hu339 (PiMM genotype) are immortalized cell lines from human liver. Although these cell lines sufficiently proliferate, they display a mesenchymal phenotype, express low levels of AAT, and do not aggregate AAT compared with Huh 7.5 cells; Huh 7.5 cells are a hepatocyte-derived cellular carcinoma cell line that the good levels of hepatocyte-specific functions (Fig 3A). We used AT01 and Hu339 as a genotypically PiZZ and PiMM scaffold for studying the effects of MAAT and ZAAT in transient transfection studies.

To determine the role of SVIP in AATD, first we examined the expression level of SVIP in normal and AATD human liver tissues and Hu339 and AT01 cell lines by qPCR and IB. The accumulation of misfolded ZAAT using pTR2-CB-ZAAT in the ER of the Hu339 cell line significantly upregulated SVIP; this was comparable to AT01 cells, which show an elevated level of SVIP protein. This result is consistent with a previous report that ER stress upregulates SVIP, which is expected to inhibit ERAD [33]. Inducing ER stress in Hu339 cells using pTR2-CB-ZAAT 48 hours post transfection significantly upregulated SVIP, which has no effect on the p97/VCP expression level (Fig 3B). To evaluate SVIP expression in liver tissues quantitatively, we separately ground frozen normal and AATD liver tissues in liquid nitrogen and subjected them to qPCR or IB analysis. The expression of SVIP protein was markedly upregulated in deficient individual liver tissues as compared with the normal group (Fig 3C).

In our real time qPCR analysis, SVIP mRNA expression was relatively high in the AT01 cell line and Hu339 transfected with ZAAT, compared with control Hu339 samples (Fig 3D). SVIP mRNA expression was relatively high in AATD individuals, which is consistent with the immunoblot results (Fig 3E).

### SVIP negatively regulates gp78-VCP mediated ERAD

**Fig 4:**
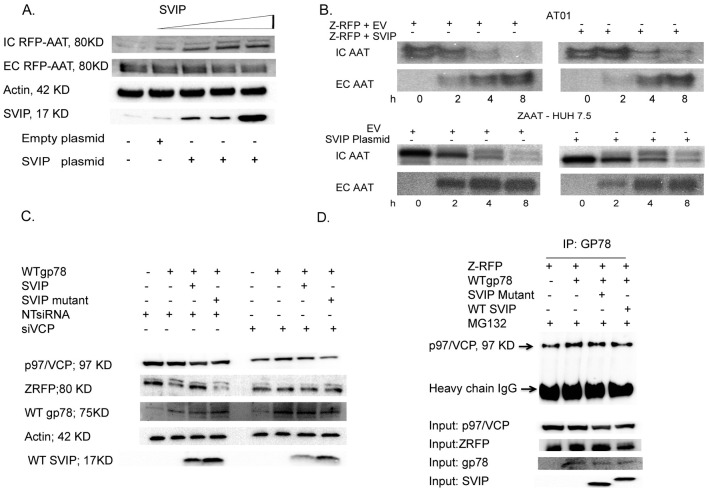
SVIP inhibits ERAD. (A) SVIP caused dose-dependent accumulation of ZAAT. AT01 cells were transfected with ZAAT-RFP and increasing amounts of PCMV6-entry-SVIP (0, 1, 2, 4 μg). The levels of AAT were determined by Western blotting with anti-AAT antibody. Actin was blotted as a loading control. (B) SVIP overexpression inhibited ZAAT degradation. AT01 and ZAAT-expressing stable Huh cells were co-transfected with Z-RFP and either with empty plasmid or PCMV6-entry-SVIP (2 μg). At 48 hours after transfection, the cells were radiolabeled with 500 μCi ^35^S and chased for the indicated times. The levels of intracellular (IC) AAT were determined by SDS-PAGE autoradiography. (C) SVIP negatively regulates gp78-p97 mediated degradation of AAT. ZAAT-expressing stable Huh cells were transfected with NTsiRNA or siVCP/p97. 24h post silencing they were co transfected with gp78, gp78 and WT SVIP or SVIP mutant. The IC ZAAT, VCP, gp78 and SVIP were detected by western blott. Actin was used as loading control. (D) SVIP inhibited gp78-p97/VCP interaction. AT01 cells were transfected as indicated before and were processed for IP with anti-gp78 antibody for endogenous gp78. p97/VCP bound to gp78 was detected by Western blotting with anti-VCP antibody.

Overexpression of SVIP induces cellular vacuolation, which represents enlarged ER [41]. There are some studies that show such vacuoles might be caused by the accumulation and aggregation of misfolded proteins within the ER and the inhibition of ERAD due to overexpression of SVIP [30]. To test the relation between SVIP overexpression and increased ZAAT accumulation, we performed experiments to detect the effects of SVIP on ERAD. First, we transfected the AT01 cell line with the ZAAT-RFP plasmid. At 24 hours post transfection, different doses of SVIP WT plasmid were introduced to the cells. SVIP caused a dose-dependent accumulation of ZAAT in AT01 cells. There was no significant change in the extracellular level of AAT; thus, ZAAT accumulation is due to the inhibition of degradation (Fig 4A).

Then a similar experiment was performed to evaluate the level of degradation by ^35^S pulse chase analysis in AT01 cells transfected with the Z-RFP plasmid with overexpression of SVIP, as well as stable RFP-ZAAT–expressing Huh 7.5 cells. Overexpression of SVIP significantly inhibited ZAAT degradation in both cell lines with no significant changes in extracellular level of ZAAT (Fig 4B). To determine the inhibitory role of SVIP on gp78-p97/VCP mediated degradation of ZAAT, we used NTsiRNA as control and siVCP to silence p97/VCP in ZAAT-expressing stable Huh cells. 24 hours post silencing, the cells were co-transfected with plasmids encoding WT gp78, WT gp78 and WT SVIP or mutant SVIP (VIMm). The level of IC ZAAT was detected by western blot using anti-AAT antibody. The results show that in presence of NT siRNA, gp78 overexpression enhances ZAAT degradation. Only WT SVIP can inhibit gp78-mediated ZAAT degradation and not SVIP mutant. In the cells wich p97/VCP was knocked down, IC ZAAT remains stable and non of the treatments can affect IC ZAAT level. These results indicate that the inhibitory role of SVIP on gp78- mediated degradation of AAT depends on p97/VCP and si p97/VCP stabilizes IC ZAAT (Fig 4C). To determine the effect of SVIP on gp78-p97/VCP interaction we performed an experiment. AT01 cells were transfected with 2 μg Z-RFP plasmid. At 24 hours post transfection, they were co-transfected with plasmids encoding WT gp78, WT gp78 and WT SVIP or mutant SVIP (VIMm). Then the cells were treated with proteasome inhibitor MG132 to block p97/VCP-mediated degradation of ZAAT were processed for IP for gp78, followed by Western blotting for p97/VCP. The results show that the overexpression of WT SVIP inhibits p97/VCP binding to gp78 (Fig 4D).

### Endogenous SVIP inhibits ZAAT degradation

**Fig 5:**
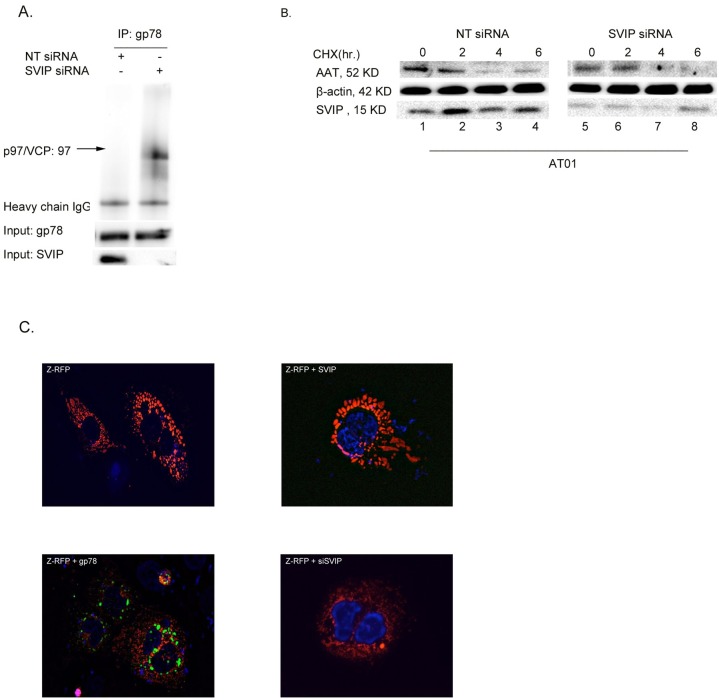
Endogenous SVIP inhibits gp78-p97/VCP interaction, which leads ZAAT accumulation. (A) Endogenous SVIP inhibits gp78-p97/VCP association. gp78 was immunoprecipitated from AT01 transfected with non targeting siRNA or siSVIP (2 μM). p97/VCP associated with gp78 was determined by IB with anti-p97/VCP antibody. (B) Silencing SVIP expression increased ZAAT degradation. AT01 cells were transfected with negative siRNA as control or SVIP siRNA. At 72 hours after transfection, ZAAT degradation was tested with a 20-μg/mL cycloheximide (CHX) chase. SVIP was blotted with polyclonal anti-SVIP antibody. (C) Immunofluorescence microscopy for Z-AAT in Huh knockout cells 48 hours post transfection with ZAAT-FRP with or without EGFP-gp78, SVIP plasmid, and siSVIP. Z-AAT is shown in red, and gp78 is shown in green. DAPI-stained nuclei are shown in blue.

Our data further support the hypothesis that endogenous SVIP negatively regulates the function of gp78 in the hepatocytes with ZAAT aggregation. To determine whether endogenous SVIP diminishes gp78 interaction with p97/VCP, we silenced the expression of SVIP and examined the association of gp78 and p97/VCP by co-IP in the AT01 cell line. The silencing of SVIP significantly induced the interaction of p97/VCP with gp78, which is consistent with the silencing of SVIP inducing ERAD (Fig 5A).

Next, using AT01 cells, we investigated whether endogenous SVIP inhibits gp78-mediated ERAD. siRNA was used to silence endogenous SVIP expression. Degradation of ZAAT was detected by cycloheximide chase analysis. As expected, downregulating the SVIP expression reduced ZAAT accumulation, as reported previously [30], time dependently (Fig 5B); this was confirmed by immune flourscent microscopy (Fig 5C). All together, these results suggest that SVIP overexpression acts as an endogenous inhibitor for gp78-mediated ERAD, which acts through its common VIM with gp78 in cell lines under prolonged ER stress; furthermore, changes in the level of SVIP and gp78 proteins may control the efficacy of ERAD.

### Vacuolating phenotype of AT01 caused by SVIP overexpression

**Fig 6:**
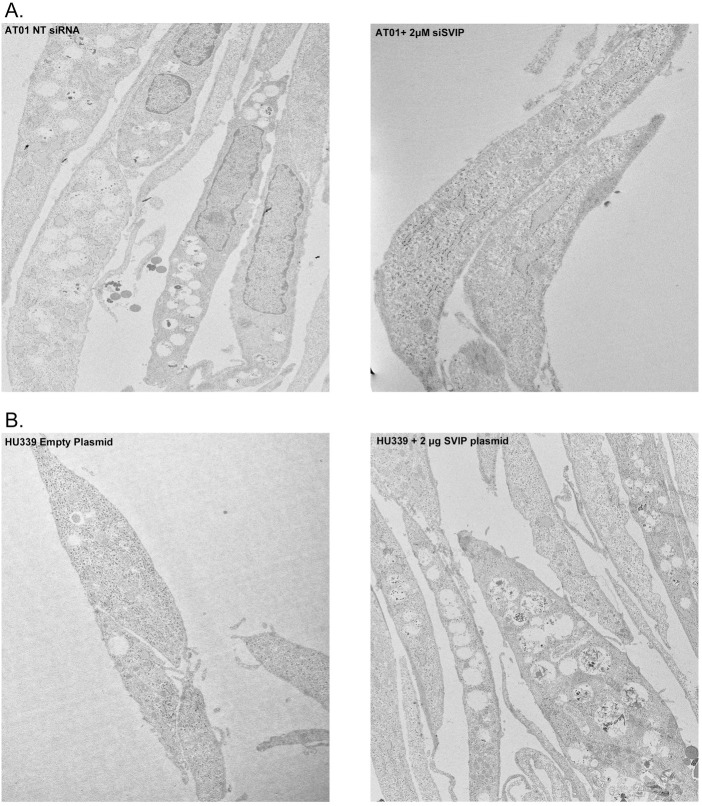
SVIP overexpression induces vacuolization of cells. **(A) AT01 cells were treated with** NT siRNA or 2 μM SVIP siRNA for 48 hours. siSVIP diminishes the SVIP-induced vacuoles in AT01 cell line. (B) vacuolization in the Hu339 cell line transfected with 2 μg SVIP plasmid.

The overexpression of SVIP has been previously shown to cause vacuole formation, which was previously hypothesized to be enlarged ER structures [42]. These vacuoles were reminiscent of cytopathic effects induced by abnormal protein aggregates [41, 43]. Because AT01 cells overexpress SVIP, we hypothesized that AT01 cells would also display the vacuolating phenotype as a consequence of this overexpression, which should not exist in the Hu339 cell line due to its lower expression level of SVIP (Fig 3). Indeed, by electron microscopy, we observed dramatic vacuolation of AT01 cells that is abrogated by the silencing of SVIP (Fig 6A).

Next, we overexpressed SVIP in Hu339 cells and also observed vacuolation in this cell line. Hu339 cells were treated with 2 μg of empty plasmid or SVIP plasmid. We processed cells for electron microscopy 48 hours after transfection. As shown in Fig 6B, vacuoles were frequently observed in the cytosol of Hu339 cells treated with 2 μg SVIP plasmid, as compared with control Hu339 cells.

## Discussion

Z mutation is a Glu342Lys that results in misfolding and an abnormal tendency of ZAAT to aggregate within the ER of hepatocytes, causing a toxic gain of function [40]. Previous studies have suggested that ERAD is involved in ZAAT degradation [44]. Using direct fusion of ERAD substrates with GFP as ERAD reporters, each substrate uses a unique set of different ERAD components, which is substrate specific for degradation [45]. gp78 is an E3 ubiquitin ligase that integrates ERAD by recruting p97/VCP for retro translocation. A growing list of substrates including ZAAT has been identified for gp78, highlighting the importance of gp78-mediated ERAD in physiological pathways [46]. ERAD pathways include several components, such as ER resident E3 ligases and the p97/VCP AAA ATPase [47, 48]. The p97/VCP-gp78 complex participates in the final step of ERAD. The gp78-p97/VCP complex acts as a scaffold protein to assemble a complex that couples ubiquitination, retro-translocation, and degradation [49, 50]. gp78 E3 ligase targets a variety of misfolded ERAD substrates for degradation via interaction with p97/VCP [27, 51, 52]. Consistent with previous reports [23], our data confirm the involvement of gp78 in ZAAT ERAD. We showed that gp78 overexpression targets ZAAT for proteasomal degradation in a hepatocyte-like ZZ cell line and ZAAT-expressing Huh cells. These cell lines naturally have the AAT-specific ERAD machinery, which make them more appropriate to study AAT trafficking, compared with HEK293 or CHO cells, which have traditionally been used for this type of studies.

To confirm the role of p97/VCP in ZAAT degradation, we transfected AT01 cells with a modified form of gp78 without the VIM (gp78 Δ135) compared with WT gp78. The gp78 mutation in the VIM domain could not enhance clearance of ZAAT aggregates. Moreover, p97/VCP co-immunopreciptates with ZAAT in the presence of WT gp78 but not gp78 Δ135. This result sugests that gp78 and p97/VCP are involved in ZAAT degradation and the VIM motif of gp78 is necessary to form the complex.

In this study, we also demonstrated that SVIP, a previously identified p97/VCP-interacting protein, negatively regulates gp78-mediated ZAAT degradation. This report may reveal a few clues with respect to ZAAT degradation. Our current study using liver tissue samples shows higher levels of SVIP in liver samples from AATD individuals, as well as in a PiZZ liver cell line. This phenotype is seen in the cells under prolonged ER stress. SVIP is an endogenous ERAD inhibitor [30] expressed in high levels in ZAAT cell lines and in human PiZZ liver tissues. SVIP may protect liver cells from damage that enhanced ERAD may cause under prolonged ER stress conditions [53]. According to previous reports, changes in the levels of SVIP and gp78 proteins control the efficacy of ERAD in 293 cells [30]. We wanted to determine what mechanism underlies this regulatory pathway in cells that naturally have the secretion machinery for AAT, as well as in liver tissue from PiZZ and PiMM individuals. Our study shows that SVIP overexpression causes a ZAAT degradation delay in AT01 cells, as well as in a ZAAT transfected Huh339 cell line. To explain this, we performed several experiments to investigate the link between SVIP and ZAAT degradation. We found that gp78 and p97/VCP interact in liver tissue samples from AATD individuals. However, these individuals still have large amounts of aggregated AAT. These findings led us to believe that the intensity of the gp78 interaction might be insufficient to extract misfolded ZAAT and unable to target ZAAT for proteasomal degradation. Accumulation of protein creates a vicious cycle by which ER stress elevates the SVIP protein level and elevated SVIP reduces clearance of ZAAT, even though gp78-VCP association is detectable. gp78 interacts with p97/VCP through VIM, where it serves to recruit p97/VCP to the ER membrane to assist in the retro-translocation of gp78 substrates, such as the Z variant of AAT [54]. This highly conserved VIM is also found in SVIP, which introduces a possible regulatory role of SVIP on gp78-mediated ERAD. After silencing SVIP in ZAAT-expressing cells, we noticed an induction of p97/VCP-gp78 association. This enhanced association leads to reduction of aggregated ZAAT inside the ER. By analyzing ZAAT as a gp78 substrate, we showed that enhancing gp78-p97/VCP interaction enhances ZAAT association. ZAAT-p97/VCP interaction may reduce the accumulated ZAAT inside the ER by facilitating proteasomal degradation of ZAAT in hepatocytes.

In addition, overexpressing SVIP reduces clearance of ZAAT, and knocking down SVIP increases ZAAT clearance in presence of p97/VCP. Inhibition of p97/VCP by siRNA also stabilizes ZAAT. Therefore, we demonstrate that clearance of ZAAT can be accomplished by overexpressing gp78 or by knocking down SVIP in the presence of functional p97/VCP.

SVIP causing vacuolation is a very interesting cytopathic effect [41]. In a previous report, the reduction of VCP levels induced vacuolization of the cytoplasm as a consequence of ER expansion. Ribosomes are frequently detectable on the cytosolic side of these ER-derived vacuoles [55]. The formation of large vacuoles may be a result of insufficient p97/VCP available for gp78 to mediate retro-translocation as a consequence of SVIP overexpression [43]. Because AT01 (ZAAT) cells have elevated SVIP compared with Hu339 (MAAT) cells, we tested them for the presence of vacuoles as an indication of SVIP overexpression in AT01. We believe prolonged ER stress causes morphological alterations that accompany the aggregation of ZAAT and overexpression of SVIP. Hu339 cells have lower SVIP and do not display a vacuolization phenotype. Although there are no available data for the mechanism underlying vacuolation [41], our data are consistent with previous studies and show that silencing SVIP as an ERAD inhibitor could reduce this morphological change in AT01 cells. A possible explanation for the presence of vacuoles is that the aggregation of ZAAT molecules is toxic and cells only survive if they are capable of engulfing them within a membranous subcompartment [56].

In the present study, we demonstrate that the high level of SVIP competes with gp78 to interact with p97/VCP, attenuates the interaction of gp78 and p97/VCP, and inhibits ZAAT retro-translocation and ERAD. In summary, our results demonstrate that SVIP may be a regulator of the gp78-mediated ubiquitin-dependent degradation of ZAAT in hepatocytes. We propose that p97/VCP can form a complex with either gp78 or SVIP. SVIP is a negative regulator of gp78-p97/VCP–mediated ERAD. We believe that the misfolded and aggregated ZAAT affects the stoichiometry between these complexes and establishes a positive feedback cycle that reduces degradation of ZAAT. As intracellular ZAAT inclusions are believed to be hepatotoxic and the main cause of early liver disease in PiZZ individuals, clarifying the molecular mechanisms associated with gp78-mediated ERAD may lead to the identification of novel therapeutic targets.

## Compleance with ethic guidlines

Nazli Khodayari, Rejean liqun Wang, George Marek, Karina Krotova, Mariana Kirst, Chen Liu, Farshid Rouhani, and Mark Brantly declare that they have no conflict of interest.
